# Policy Challenges Facing the Scale Up of Integrated Community Case Management (iCCM) in Uganda

**DOI:** 10.34172/ijhpm.2021.39

**Published:** 2021-05-24

**Authors:** Phyllis Awor, Joan Nakayaga Kalyango, Cecilia Stålsby Lundborg, Freddie Ssengooba, Jaran Eriksen, Elizeus Rutebemberwa

**Affiliations:** ^1^School of Public Health, Makerere University College of Health Sciences, Kampala, Uganda.; ^2^Clinical Epidemiology Unit, College of Health Sciences, Makerere University Kampala, Kampala, Uganda.; ^3^Department of Pharmacy, College of Health Sciences, Makerere University Kampala, Kampala, Uganda.; ^4^Health Systems and Policy, Department of Global Public Health, Karolinska Institutet, Stockholm, Sweden.; ^5^Unit of Infectious Diseases/Venhälsan, Södersjukhuset, Stockholm, Sweden.

**Keywords:** Community Case management, Malaria, Pneumonia, Diarrhoea, iCCM Policy Analysis, Uganda

## Abstract

**Background:** Integrated Community Case Management (iCCM) of malaria, pneumonia and diarrhoea is an equity focused strategy, to increase access to care for febrile illness in children under-5 years of age, in rural communities. Lay community members are trained to diagnose and treat malaria, pneumonia and diarrhoea in children, and to identify and refer very ill children. Today, many low-income countries including Uganda, have a policy for iCCM which is being rolled out through public sector community health workers (CHWs). Ten years after the introduction of the iCCM strategy in Uganda, it is important to take stock and understand the barriers and facilitators affecting implementation of the iCCM policy.

**Methods:** We conducted an iCCM policy analysis in order to identify the challenges, enablers and priorities for scale-up of the iCCM strategy in Uganda. This was a qualitative case study research which included a document review (n=52) and key informant interviews (n=15) with Ugandan stakeholders. Interviews were conducted in 2017 and the desk review included literature up to 2019.

**Results:** This paper highlights the iCCM policy trajectory since 2010 in Uganda and includes a policy timeline. The iCCM policy process was mainly led by international agencies from inception, with little ownership of the government. Many implementation challenges including low government funding, weak coordination and contradicting policies were identified, which could contribute to the slow scale up of the iCCM program. Despite the challenges, many enablers and opportunities also exist within the health system, which should be further harnessed to scale up iCCM in Uganda. These enabling factors include strong community commitment, existing policy instruments and the potential of utilizing also the private sector for iCCM implementation.

**Conclusion:** The iCCM program in Uganda needs to be strengthen through increased domestic funding, strong coordination and a focus on monitoring, evaluation and operational research.

## Background

**Key Messages**

**Implications for policy makers**

- Ten years after the introduction of the integrated Community Case Management (iCCM) strategy in Uganda, it is important to take stock and understand the barriers and facilitators affecting the implementation of this policy.
- This paper highlights the iCCM policy trajectory since 2010 in Uganda, including a policy timeline.
- Overall, there has been slow scale up with coverage of 58% of districts, due to many implementation challenges which include low government funding and weak coordination and supervision. Despite the challenges, many enablers and opportunities also exist within the health system, which should be further harnessed to scale up iCCM in Uganda. These enabling factors include strong community commitment, existing policy instruments and the potential of utilizing also the private sector for iCCM implementation.
- The iCCM program in Uganda needs to be strengthened through increased domestic funding, strong coordination and a focus on monitoring, evaluation and operational research.

**Implications for the public**

In order to ensure that children with malaria, pneumonia and diarrhoea in rural communities can receive prompt diagnosis and treatment, community health workers (CHWs) are trained and equipped to provide this healthcare. This is through the scale up of the integrated community case management (iCCM) strategy in Uganda and other low-income countries. Ten years after the roll-out of the strategy, this research reviewed the available evidence including interviews with stakeholders, to identified challenges, enablers and priorities for improving access to healthcare for children in Uganda. Many challenges including low government funding and poor coordination and supervision have affected wider implementation of the CHW program. If these challenges are addressed, more children with fever, malaria, pneumonia and diarrhoea will be able to receive adequate and timely treatment, and they will be healthier, with less deaths due to these illnesses.

Every year, nearly 6 million children under five years of age die, with 95% of these deaths occurring in low- and middle-income countries.^1,2^ One half (50%) of all deaths in children 1-5 years of age in sub-Saharan Africa are caused by three diseases, namely malaria, pneumonia and diarrhoea.^3^ These three diseases usually present with similar and overlapping symptoms including fever, fast or difficult breathing and diarrhoea.^4^ Access to preventive care and timely treatment for these illnesses is important, in order to prevent unnecessary child deaths.

 Integrated Community Case Management (iCCM) of malaria, pneumonia and diarrhoea is an equity focused strategy, to increase access to care for febrile illness in children under-5 years of age, in rural communities.^5,6^ Lay community members are trained to diagnose and treat these three illnesses in children, and to identify and refer very ill children. Today, many low-income countries including Uganda^7^ have a policy for iCCM, which is being rolled out through public sector community health workers (CHWs).^8^

 The main components of the iCCM strategy include supplying CHWs with a kit of pre-packaged medicines and commodities including diagnostic tools; CHWs mobilizing communities to demand, support and use the iCCM intervention; CHWs treating children under five with fever, cough and diarrhoea and counselling mothers on home care and care seeking; CHWs referring immediately newborns with danger signs and severely ill children and giving pre-referral rectal artesunate for severe malaria; CHW collecting iCCM data and reporting timely; peer supervision amongst the CHWs; and trained health facility staff managing referred cases and supervising CHWs in their catchment area and monitoring program progress.

 The positive effect of the iCCM interventions on access, quality of care and morbidity, are well-documented within the public sector.^5,9^ Where CHWs are well-trained; supervised; and supplied with the drugs and commodities, the iCCM intervention may reduce mortality in children.^10,11^

 In 2010, iCCM was introduced in Uganda with the launch of the iCCM strategy and with the release the national iCCM implementation guidelines and training manuals. About 10 years later, we set out to identify enablers, challenges and priorities for scale-up of the iCCM strategy in Uganda.

## Methods

###  Design

 We conducted a qualitative case study research which included a document review (n = 52) and key informant interviews (n = 15) with Ugandan stakeholders. Interviews were conducted in June and July 2017 and the desk review included literature up to 2019.

###  Data Collection 

 The document review included a search of iCCM literature from peer reviewed and grey sources including government policies, guidelines, directives and implementing partner reports, as well as a synthesis of discussions from the Ministry of Health’s (MoH’s) iCCM Technical Working Group meetings during the study period. The document review enabled us to create a detailed timeline of iCCM related policy development in Uganda, as well as an initial list of respondents for the key informant interviews.

 Key informant interviews were conducted with national iCCM stakeholders including policy-makers, program managers and implementing partners who were identified based on their current position and from the document review. All organizations currently supporting the implementation of iCCM in Uganda were included, contacted and requested for interview. A snowball approach was also used to find more interviewees by asking about additional stakeholders who should be interviewed. All interviewees were high level technical experts in their organization. An interview guide was developed drawing on the key concepts from the health policy analysis triangle framework by Walt and Gilson 1994.^12^

 The interviews were recorded and transcribed verbatim by two data collectors.

###  Stakeholder Analysis and Position Mapping

 In order to know the stakeholders and understand their voice in the iCCM policy process, we conducted a stakeholder analysis. This was also informed by the key informant interviews and the literature review. We also conducted position mapping of stakeholders as recommended by Buse et al^13^ by: identifying the policy actors, assessing their power or political resources, and understanding their position and interest with respect to the policy. This map includes the stakeholders listed against their position of influence and colour coded based on their power - See the section on Actors. The judgment of opposition or support were mainly arrived at through interpretation of the power and interest of actors. Power (the ability to make decisions, control resources and influence people) was conceptualized based on both individual ability (agents) and structures or organizations to which they belong. This focused on those individuals and organizations who make and implement iCCM policy decisions, looking at both inputs and outputs in the policy-making and implementation.

###  Data Analysis

 We conducted a retrospective analysis of the policy and described the current status of iCCM implementation.

 The study and analysis were guided by the Health Policy Analysis Triangle conceptual framework^12^ as a starting point, which was complemented by the modified stages heuristic public policy framework,^14-16^ see Figure (a and b). The policy analysis triangle framework focuses on policy change as being shaped by the policy content, context and process, as well as the existing interactions between these dimensions. While this policy analysis triangle was a useful starting point, it did not adequately inform the complexity of the policy uptake, implementation and sustainability.^17^ The modified stages heuristic policy framework^14^ in Figure (b) better depicts the turbulent nature of the policy process and informed analysis of additional factors relevant for wide scale policy implementation and sustainability, including evaluation and factors affecting policy adoption. These were coded and reported as challenges, enablers and opportunities for policy uptake^18^ in the analysis.

- Under context: we analyse and present the iCCM policy development timeline and trajectory over 20 years, as well as describe political and social factors of influence.
- Under content: we review and describe the substance of the iCCM policy.
- Under actors: we identify the organization and persons and then assess their power and position in influencing the policy process and uptake.
- Under process, adoption and implementation (informed by both the analytical models): we discuss the early adoption process and the extent of uptake of the policy and implementation after ten years. This includes any evaluations undertaken, the challenges, enablers and opportunities that exist.

**Figure:**
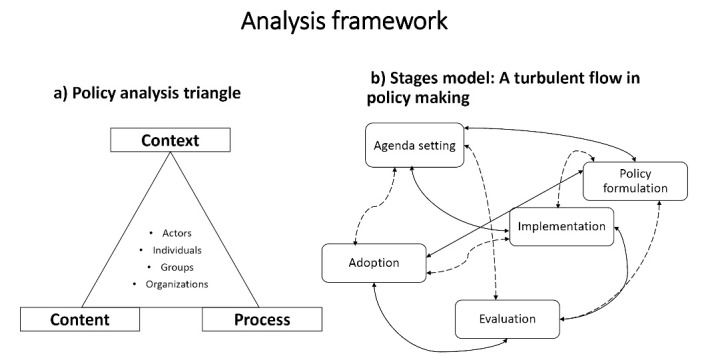
Analysis Framework.

 The interviews were analysed by the first author and two members of the research team (see Acknowledgments), using manifest content analysis.^19^ After the interviews, analytical codes were developed within the following broad categories: *policy content, context, process, implementation, challenges,*and*enablers*.

 For the document review, a document library was created and then reviewed to draw out issues related to the development and implementation of the iCCM policy.

 We triangulated across respondents and between interviews and the document review during analysis, to corroborate findings.^20^ We also held a validation workshop to share the draft report with stakeholders and to obtain feedback on the emerging findings. Analysis led to identification of an additional policy relevant theme namely:*opportunities for scale up*of iCCM. Results have therefore been structured according to 7 thematic categories which include the 6 pre-determined codes which were also analytical themes as well as the seventh theme (opportunities for scale up of iCCM).

## Results

###  Data Sources

 A total of 52 documents were reviewed including policy documents, strategic plans, training manuals, peer reviewed and other grey literature. Fifteen key informant interviews were also conducted. The key informants represented the following organizations, all of whom are currently supporting the implementation of iCCM: MoH; The AIDS Support Organization; United Nations Children’s Fund (UNICEF); World Health Organization (WHO); Malaria Consortium; Plan international; Pilgrim; International Rescue Committee; Uganda Health Marketing Group; Save the Children; Clinton Health Access Initiative; Living Goods; and the Program for Accessible Communication and Education. All the organizations included lead significant work on iCCM in different regions of the country. Table 1 provides an overview of the data sources.

 A total of 5 key informants were involved with the iCCM policy since its inception in Uganda. Two were directly involved in the policy design and 3 with the implementation.

**Table 1:** Overview of Data Sources

**Documents and Key Informant Interviews**	**Number**
Number of documents reviewed	52
Number of key informant interviews completed by category	
Government officials (MoH)	2
Multilateral agencies eg, WHO and UNICEF	3
NGOs including national and international	10
Total respondents interviewed/approached	15/18

Abbreviations: MoH, Ministry of Health; UNICEF, United Nations Children’s Fund; NGOs, non-governmental organisations; WHO, World Health Organization. *Note*. We were not able to interview 1 government official and 2 NGO participants after 3 attempts.

###  Context

 iCCM is not a stand-alone policy but part of the integrated management of childhood illnesses (IMCI) program, as the community level implementation of IMCI. Table 2 shows an abbreviated timeline of iCCM policy development in Uganda. The timeline starts with adoption of the WHO promoted IMCI strategy in 1995, in Uganda. The IMCI strategy aimed to reduce child mortality and morbidity in developing countries, by combining improved management of childhood illnesses with proper nutrition. The IMCI strategy includes 3 main components: improving facility level case management, improving overall health systems, and improving family and community health practices. Evaluation of the IMCI policy from 2000-2005 showed poor performance, mainly due to weak implementation of the community component and lack of access to health facilities by poor families with sick children. In order to address these community-based challenges, the iCCM strategy was adopted in Uganda and other developing countries. After the year 2010, various Uganda health sector strategies and plans recognize iCCM as a key child survival strategy – see Table 2.

**Table 2:** Abbreviated Timeline for iCCM Policy Development in Uganda

**Date**	**Event**	**Reference (Document or Interview ID)**
1995	IMCI strategy adopted in Uganda (1995)	Uganda MoH, 1995^24,25^
2000	IMCI operational and scaled to across the entire country; and incorporated into the first Uganda health sector strategy: Health Sector Strategic Plan I (1999/2000–2004/2005)	Uganda MoH, 2000^26^; UG05 – Government official
2004	IMCI national evaluation – showed absolute levels of quality of care remained low; and weak development of the community IMCI component	Sabiiti et al, 2004^22^; Pariyo et al, 2005^21^
2005	Pilot projects showing feasibility of community management of malaria; and a review of home-based management of fever strategy	KI 04 (multilateral agency)^27-29^
2005	Health sector strategic plan II (2005/2006–2009/2010) – incorporated iCCM as the community component of IMCI	MoH 2005^30^
2008	Global – iCCM strategy	WHO/UNICEF joint statements 2008 and 2012^31^
2009	Child survival strategy 2009/2010–2014/2015; A 5-year national strategic plan for accelerated child survival and development in Uganda – incorporates iCCM for improved child survival	MoH, 2009^32^
2010	VHT strategy	MoH, 2010^33^
2010	Health sector strategic plan III 2009/2010–2014/2015	MoH, 2010^34^
2010	Uganda iCCM implementation guides; Launch of iCCM in Uganda	MoH, 2010^7^; KI 04 multilateral agency
2013	Reproductive, maternal, newborn, child and adolescent health sharpened plan: a promise renewed	MoH, 2013^35^
2014	Uganda iCCM implementation plan	MoH, 2014^36^
2014	Start of the national iCCM coordination mechanism – technical working group and regular meetings	KI 03, Government official
2015	Uganda Malaria Reduction Strategic Plan – 2015-2020	MoH, 2015^37^
2017	CHEW strategy	MoH, 2017^38^

Abbreviations: IMCI, integrated management of childhood illnesses; MoH, Ministry of Health; VHT, village health team; iCCM, integrated Community Case Management; CHEW, Community Health extension worker; UNICEF, United Nations Children’s Fund; WHO, World Health Organization; KI, key informant.

 The iCCM got on to the health policy agenda in Uganda through strong support from international agencies. A policy window opened internationally when it was clear that many low-income countries were off track and likely not to achieve the Millennium Development Goal 4, on child health. In addition, there was local and global evidence showing that the IMCI strategy was performing poorly, partly due to weak community level implementation.^21,22^ In Uganda there was also evidence of success from the home-based management of Malaria program where 60% of febrile children were reached with malaria medications at home; and evidence from an iCCM pilot in post conflict northern Uganda which showed high acceptability and feasibility of implementing iCCM.^23^

###  Content

 As mentioned, iCCM is the community level component of another policy on IMCI.^24^ Existing iCCM policy instruments include: the national iCCM strategy, implementation plan, guidelines and reporting tools.^7^ iCCM is operationalized through the village health team (VHT) strategy in Uganda, which mandates lay community volunteers to participate in health promotion and care. With iCCM, lay community members are trained to diagnose malaria (using the malaria rapid diagnostic test), pneumonia and diarrhoea in children under five years of age and to treat appropriately with artemisinin combination therapy, amoxicillin and oral rehydration salts and zinc tablets, respectively.

 The contents of the VHT kit for iCCM include: Pre-packaged medicines for malaria, pneumonia and diarrhoea including amoxicillin for non-severe pneumonia, ACT for uncomplicated malaria, low-osmolarity oral rehydration salts for diarrhoea, zinc for diarrhoea and rectal artesunate for pre-referral treatment of patients with severe malaria; diagnostic commodities eg, malaria rapid diagnostic test, respiratory timers, mid-upper arm circumference tape; and user items eg, job aid cards.

 iCCM is recognized as a key strategy for increasing treatment coverage for the main causes of childhood mortality in other policy documents including the Reproductive Maternal Newborn Child and Adolescent Health Sharpened Plan; the Uganda Malaria Reduction Strategic Plan; and the national new born and child survival strategies.

###  Actors

 The key actors in this policy formulation and process were from International and local, bilateral agencies, the MoH, ministry of finance, research institutions, academia, consultants, Health professional organizations and individual doctors and nurses.

 The iCCM policy formulation was generally led by international partners supporting the MoH technocrats to adopt the global iCCM strategy for the Ugandan context. The main proponent agencies were UNICEF and WHO and other implementing partners participated including Save the Children and Malaria Consortium. These generally had high power and high influence over the process. The international actors also supported the MoH to develop the iCCM implementation guidelines, facilitator guide, and implementation plan. However, to date, there has been no high level policy champion for iCCM.

 There was some initial resistance from various stakeholders. First, the low education level of the lay cadre of volunteer CHWs was of concern particularly to paediatricians and some other professional health associations and workers. Secondly, the proposed distribution of pneumonia medicines (amoxicillin) at community level was resisted by the MoH pharmacy department, which was concerned about antimicrobial resistance. Numerous evidence sharing workshops which included presentations from international experts helped to reduce the initial resistance and to create consensus.

*“…it was a whole year of consultation. We had so many stakeholder meetings at Hotel Africana because there was some resistance”* (KI 4, Donor/Multilateral agency).

 While paediatricians, professional associations and some doctors provided the highest resistance to the policy, these generally have low power to influence change. The medium opposition from Ministry of Finance, which also has high influence, is due to their lack of available financing for the program. Table 3 presents a stakeholder map of the position and power of actors, in relation to the iCCM policy.

**Table 3:** Position and Power of Stakeholders in Relation to the iCCM Policy

**High Opposition**	**Medium Opposition**	**Low Opposition**	**Neutral**	**Low Support**	**Medium Support**	**High Support**
Medical associations, paediatricians, MoH- Pharmacy department	Ministry of Finance			District health teams	DGHS, MoH-Child health department, Malaria Control Division	International NGOs (UNICEF, WHO, Save the Children)

Abbreviations: MoH, Ministry of Health; DGHS, Director General of Health Services; UNICEF, United Nations Children’s Fund; WHO, World Health Organization; NGOs, non-governmental organisations; iCCM, integrated Community Case Management. Colour code (from darkest to lightest): Black represents highest power and influence.

###  Process

 The iCCM policy adoption was slow from the start and heavily dependent on the contextual factors and actors described earlier. While there were credible actors like the international agencies leading the process, the lack of national high level political commitment, and unstable funding affected wide scale implementation. The policy process is discussed in more detail under the sections of implementation, challenges, enablers and opportunities below.

###  Implementation of iCCM in Uganda

 Public sector coverage of iCCM implementation is currently 75/130 (58%) districts in the country, less than the targeted 90% of districts by 2018 [Uganda Malaria Reduction Strategic Plan, 2015; and communication from the iCCM program].

 Early implementation of iCCM started in hard-to-reach regions and high mortality areas of the country, including within the post conflict northern Uganda. Implementation is mainly donor/partner funded with the government of Uganda contributing about 16% of the total iCCM budget in 2016/2017 [iCCM annual program data]. Key funders include the Global Fund, the UK Department for International Development (DfID) and UNICEF. Other key stakeholders include the National Medical Stores and its supply chain division, whose responsibility is to ensure that medicines reach the last mile health facilities and eventually the CHW.

 In terms of the effectiveness of implementation in Uganda, the introduction of iCCM has contributed to increased access to treatment by children at community level and to reduced outpatient attendance or patient load.^39^ However, operational research and program evaluation have not been fully embedded into the iCCM program and there is no impact level data for Uganda. In addition, various enablers and challenges affect iCCM implementation and scale-up. These are discussed in the next section.

*“ iCCM may not have had much impact because of that scale at which it has been implemented. I wish we had implemented throughout the country, it would have had a great impact. I believe that the reduction of child mortality are from 54 to 43 partly has been due to iCCM but also due to other interventions like net distributions through mass campaigns and antenatal care” *(KI 13, MoH official).

*“We would like to see academia doing for us a study so as to understand the impact of iCCM and how much we can get out of iCCM programs so that it also acts as an advocacy and hence more funding for the iCCM programs” *(KI 1, Donor).

###  Challenges Affecting the Implementation and Scale Up of iCCM in Uganda

 We identified numerous barriers and challenges affecting the implementation of iCCM in Uganda. Many of these challenges pose threats to the scale up of the policy. A key challenge relates to underfunding and unsustainable funding for iCCM. Most funding for iCCM is from donors and implementing partners, with minimal government/Ministry of Finance budgetary support for the iCCM program. In 2016/2017, budgetary support to iCCM was only 16% of the total funding required by the program. Even with donor support, the iCCM program is hugely underfunded [iCCM annual program data]. There is insufficient funding for the required scale-up, including for the necessary medicines and diagnostic tests, resulting in frequent stock out of the required iCCM commodities.

*“There is no public sector financing; mainly implementing partners are funding iCCM, yet also in a fragmented manner” *(KI 2).

*“The lessons we have learnt is that some of these interventions come with very good designs and strategy but lack funding and you may not realize a greater impact of such an intervention” *(KI 13).

*“You realize that iCCM commodities consume a big chunk of the iCCM program budget so you realize that there are very few donors that have been able to put in resources for implementation of iCCM programs and specifically for procurement of iCCM commodities. Therefore that has been one of the biggest barriers” *(KI 4).

 The second challenge is weak coordination of the iCCM program. The iCCM program is managed by two different departments at the MoH in Uganda. These are the child health department and the malaria control division. The program is designed like this because funding for the malaria component (from the Global Fund) is channelled ‘vertically’ through the malaria control division while the other components of iCCM (pneumonia and diarrhoea) remain the responsibility of the child health department. This is a reality that has to be accommodated, but management across departments has been challenging.

 In addition, there exists an iCCM coordinating committee which is tasked with coordinating and guiding iCCM implementation nationally. However, during the study period the committee met irregularly. The coordination at the district level was also reported to be weak, as in the quotation below.

*“At the district level there is almost no committee … there is need for a focal person and there is need for a committee too. There is also need for an MOU to describe what the different actors do and the structure is then well defined but all this seems not to be there. Therefore coordination is poor” *(KI 2, MoH official).

 A third challenge is related to confusing and contradicting communication related to the community health extension worker (CHEW) strategy. The 2016 CHEW strategy originally poised CHEWs to replace the iCCM implementing volunteer CHWs.^38^ This created tension and confusion amongst funders, implementers and the districts leadership. Although clarification was made by the MoH that CHEWs and iCCM implementing CHWs will coexist and work together (with the CHEWs as the CHW/VHT supervisors), there persists some level of confusion at the implementation level. It is worth noting that to date, the CHEW draft policy has not yet been approved by parliament, due to technical reasons.

*“We are yet to conduct trainings in 11 districts. We would have finished but the Permanent Secretary issued a circular to the districts instructing them to that there should be no more training of the VHTs in preparation for the community health extension workers”* (KI 8, Implementing partner).

*“The CHEW strategy has not yet started (being implemented) but generally, the CHEW will co-exist with the VHTs because the VHTs will be the equivalent of the health army in Ethiopia because we got the two strategies from Ethiopia” *(KI 4, Donor and Implementing partner).

 Finally, data reporting from the community health system is a big challenge. Only 18% of all active VHTs/CHWs currently report data into the health management information system [iCCM annual program data]. This implies that data from the community is generally not utilized for planning and programmatic improvement.

*“The most significant barrier was acceptability of iCCM by the health workers and bio-statisticians as a national program. They thus need to get involved, other than looking at it as a parallel program where they think that they will be paid separately” *(KI 15, Implementing partner).

###  Enabling Factors Supporting the Scale Up of iCCM Implementation

 There are some strong factors enabling the implementation and scale up of iCCM in Uganda. One is the high level of participation and commitment from the community. The entire iCCM program is premised on volunteer CHWs who identify, treat and refer sick children. While the voluntarism and lack of incentives contributes to high attrition of CHWs, thousands of self-selected CHWs continue to provide services to communities (there are almost 100 000 CHWs in the country) [iCCM program data 2017-2020]. They have maintained this service delivery for nearly 10 years. Community members also appreciate the work of CHWs and the improved access to healthcare services that iCCM brings. Identifying pragmatic ways to motivate and incentivize CHWs would contribute to an even stronger program.

 Another enabling factor is that a full range of iCCM policy instruments exist. These including the iCCM strategy, implementation guidelines (2010), the implementation plan (2014), training manuals including the facilitator’s guide and various reporting tools. The availability of these documents makes it possible for the districts and implementing partners to take the guidelines and replicate the training and implementation in different parts of the country. This has also allowed many implementing partners to engage in iCCM implementation in the country.

 There is also positive procurement and supply chain management reform. This includes data utilization for quantification and central pooling of iCCM commodities which are now being distributed through the National Medical Stores to health facilities, as opposed to donors directly distributing the commodities. This is an attempt to ensure that all medicines and diagnostics are delivered at the same point in time, rather than piece meal.

 Finally, the existence of strong donor support for iCCM has been the biggest catalyst for implementation. Donors have mobilized finances and expertise to support the policy process and implementation from inception to date. This has contributed to consensus building, buy-in and uptake of iCCM in Uganda.

 See Box 1 for a summary of the challenges and enablers of iCCM implementation in Uganda.

**Box 1. Summary of iCCM Policy Enablers and Challenges Affecting Scale Up Community Health Extension Worker**

**iCCM policy enablers**

1. Existing iCCM policy instruments including the iCCM strategy, implementation guidelines (2010), iCCM facilitators’ manual, the implementation plan (2014), and reporting tools.
2. Existing iCCM coordination mechanisms at the national and district levels.
3. Community level support including availability of CHWs and appreciation from communities for the increased access to care.
4. Strong donor/partner interest and support for iCCM.
5. The government and donors are seeking various opportunities for iCCM funding including from DfID, the Global Fund New Funding Model; and from World Bank loans.
6. There is positive procurement and supply chain management reform, including data utilization for quantification; and central funding for iCCM commodities which are now being distributed through the National Medical Stores to health facilities (as opposed to donors directly distributing the commodities).
7. Private sector inclusion in iCCM implementation (early examples exist) could contribute to sustainability.

** Key challenges affecting implementation and scale up**

1. Unsustainable funding for iCCM: A key challenge is that most funding for iCCM is from donors and implementing partners, with minimal government/Ministry of Finance budgetary support for the iCCM program (16% in 2016).
2. Confusing and even contradicting communication related to the CHEW. The recent CHEW strategy (2016) and memos from the MoH originally poised CHEWs to replace the iCCM implementing /CHWs/VHT. This created tension and confusion amongst funders, implementers and the district leadership. Although clarification was made by the MoH that CHEWs and iCCM implementing CHWs will coexist and work together (with the CHEWs as the CHW/VHT supervisors), there persists some level of confusion at the district level.
3. Weak supply chain and frequent stock out of commodities especially none-malaria commodities and supplies.
4. Attrition of CHWs due to the volunteer nature of the program and lack of incentives.
5. Weak coordination at both the national and district levels.
6. Weak supervision of the CHWs due to inadequate funding and shortage of health workers to conduct the supervision.
7. Poor reporting: Reporting from CHWs into the health management information system is only 18% and so data from the community is not utilized for planning and programmatic improvement.

---------------

Abbreviations: iCCM, integrated Community Case Management; VHT, village health team; CHEW, Community Health extension worker; CHWs, community health workers; MoH, Ministry of Health; DfID, Department for International Development.

###  Opportunities to Scale Up iCCM

 Some opportunities to scale up iCCM were identified from both the interviews and document review. First, both government and donor interviewees reported that they had been seeking various opportunities for iCCM funding including from the Global Fund New Funding Model, DfID, and from World Bank loans. The Global Fund’s new funding model contributes the malaria commodities for iCCM implementation. Non-malaria medicines and commodities are obtained from other sources which should include the government of Uganda. As most of these options are external and insufficient, stakeholders indicated that there was a need for increased financial contribution also from the government.

 A second opportunity to scale up iCCM implementation is through the private sector. This emerged from both the documentary evidence and from the stakeholder interviews. There are now examples in the literature of successful inclusion of the private sector in iCCM implementation.^40,41^ These include the following models: use of private class C drug shops^42^; and use of incentivized (paid) CHWs.^43^ Evidence from research on the drug shop model shows high acceptability and exponential improvement in quality of care for sick children, when iCCM is introduced at drug shops in Uganda.^42,44^ The incentivized-CHW model is being used by Living Goods and BRAC social enterprises to provide incentives for appropriate treatment as well as mark-up payment to CHWs based on commodity sales.^43^ Given that more than half of all sick children initially receive care at drug shops^41,45^ and that the public sector iCCM model is expensive, utilizing existing drug shops models and incentivized-CHW models could contribute toward better sustainability of the iCCM program. The quotations below also highlight the opportunity of the private sector utilization for iCCM in Uganda.

*“The good thing is that in Uganda we have enough evidence which is home generated to support the scale up of iCCM through the private sector. It is not that we are borrowing from Nigeria and we say that we are out of context” *(KI 13, MoH official).

*“Especially if iCCM was introduced into the private sector – private clinics and drug shops – and there are RDTs, people adhere to RDT results and the people adhere to the drug dosage we would definitely have a strong intervention seen” *(KI 7, implementing partner).

*“Of course, money touches everything (the drugs, commodities, supervision and training). But if we have money what other things do we need? We need management, leadership, effective supervision, and effective coordination. We also need somebody to ensure that there is adherence. … If you say that here is the money, go and scale up; you will need people, supervision and leadership” *(KI 2, MoH Official).

## Discussion

 Policy analysis is important in understanding the generation, uptake and implementation of a specific policy.^12^ While existing studies have documented the status of iCCM policy in different countries,^46^ only a few including this one, go further to explore in detail how and why policy change has occurred, and what are the enabler and challenges experienced.^18^ This is also the first study documenting the iCCM policy process in Uganda.

 The main findings of this policy analysis are that: the iCCM policy process was mainly led by international agencies with little government ownership; and that many implementation challenges including weak coordination, weak supervision and low government funding contributed to slow scale up of the package of interventions. This policy analysis also identified some factors that can contribute towards sustainability including: the existing strong community commitment and an opportunity to reach more children with the iCCM strategy interventions, through the use of the private sector.

 However, some limitations of this work should be highlighted. Firstly, all the key informants interviewed were managers either of the program at national level, or managers of non-governmental organisations that were directly involved in supporting the implementation of iCCM in the country. We did not interview the district based front-line implementers. Each district has an iCCM focal person, (usually a health educator or a malaria focal person) who could have been able to highlight additional local implementation challenges. However, all the training of CHWs and support supervision of CHWs are financed and led by donor partners for example the AIDS Support Organization. These donor partners were adequately interviewed, as well as the heads of the iCCM programs at the national level. The review of both grey (including programmatic information) and published literature allowed additional insights into implementation. Secondly, due to the wide scope of the study – which included both a retrospective review of the policy process and a cross-sectional assessment of the status of iCCM implementation, – we were not able to adequately explore all aspects of the challenges identified in this study.

 The challenges identified in this study are not new. Similar studies have identified financing, availability of commodities and diagnostics, motivation of CHWs (including salaries, allowances and supervision) as key factors necessary for sustaining iCCM and CHW programs.^18,50^ Once these factors are lacking, as identified in this study, iCCM programs are generally weak.^48^

 For the case of Uganda, additional specific challenges were identified. One of these was the contradictory policy position that was introduced with the CHEW strategy, whose cadre of paid CHWs was to replace the existing volunteer CHWs. With the introduction of the CHEW strategy, there was confusion, demoralization of volunteer CHWs, and delayed implementation of iCCM activities. This goes to highlight the importance of wide scale consultation, consensus building and cohesion as policies are being modified.

 Another challenge of iCCM policy implementation that was identified through this work, is the coordination of implementation through two different departments in the MoH. While the child health department would be responsible for implementing the iCCM strategy, funding for malaria diagnostics and medicines (from the Global Fund for HIV, TB and malaria) are received and managed by the malaria control division, separately. Although this was specifically identified in this assessment of iCCM coordination in Uganda, vertical malaria programing generally affects iCCM programs in other countries, to varying degrees.^51^ The vertical program model often undermines integration of service delivery as well as overall health systems strengthening in low income settings.^52,53^

 McGorman et al^47^ presented a health systems framework which can facilitate design, implementation and evaluation of iCCM programs from the early phases, through expansion and scale up. Their framework presents benchmarks and indicators that allow for monitoring and comparison of iCCM programs. The indicators include: availability of a community case management (CCM) policy; an annual CCM costed operational plan; targeted CHWs providing CCM; medicines and diagnostic availability; treatment coverage; caregiver knowledge of illness signs; routine supervision coverage; correct case management knowledge; and existence of a national monitoring and evaluation plan for CCM. Specifically, for Uganda, there is no updated costed iCCM operational plan, there is high level of stock out of medicines and diagnostics, no national monitoring and evaluation plan for iCCM; and no evaluation of the iCCM program which would provide information on many service delivery indicators. Using this health systems framework, we can see that key benchmarks for scale up of iCCM in Uganda have not been achieved.

 This policy analysis therefore identifies 3 priority areas for scaling up iCCM in Uganda and other similar countries. Firstly, there is urgent need to increase financing of community health systems including iCCM, by national governments. Current funding for iCCM is mainly by donors and implementation partners, with minimal financial contribution by the government of Uganda. As iCCM commodities are constantly inadequate, there is need to expand the essential medicines budget to include all medicines and commodities that are needed by CHWs. More government funding for iCCM implies stronger government ownership and commitment to the program.

 Secondly, strengthening the central level coordination must be prioritized. While there is an existing iCCM coordination mechanism/group at the national and district levels, there has still been fragmented and uncoordinated implementation. Stronger coordination and partnership between the malaria control division and the hosting department is necessary, as well as stronger relationships between implementing partners and district health management teams at the lower levels.

 Finally, strengthening data collection and transfer from community programs into the central district health information system should be prioritized. It is a missed opportunity that about 80% of CHWs are not reporting data from their level to the next level [iCCM program data 2017-2020]. Better and complete information from the community level will contribute to stronger planning and implementation.

 The priority areas identified through this policy analysis, are similar to priority areas identified in other iCCM implementing countries.^48,49^ An iCCM evidence review symposium held in 2014, brought together over 400 participants from 35 countries in sub-Saharan Africa and 59 international partner organizations. This evidence review symposium identified the following priority action areas: monitoring and evaluation of iCCM programs; supervision and performance quality assurance; financing and cost-effectiveness; demand generation; and identifying potential private sector partnerships.^48^

 Bennett et al,^18^ in a policy analysis of iCCM in six sub-Saharan African countries, also concluded that high level political ownership of iCCM policies, as well as clearer strategies for ensuring long term sustainability are necessary for scale up of such policies. Notably, the sustainability of the iCCM program in Uganda remains unclear without adequate financing for the program and for community health systems.

 In conclusion, many iCCM implementation challenges were identified in this study, including low government funding and weak coordination and supervision at different levels. Many enablers and opportunities also exist within the health system, which should be further harnessed to scale up iCCM in Uganda. This paper also highlights priority areas for immediate action.

## Ethical issues

 This work was approved by the Higher Degrees Research and Ethics Committee of Makerere Unviersity School of Public Health; and the National Council for Science and Technology.

## Competing interests

 All authors declare that they have no competing interests.

## Authors’ contributions

 PA designed the study, the tools and led the research and literature reviews; all the other authors reviewed the different versions of the manuscript and approved the final version for submission.

## Funding

 We acknowledge funding and support from the Swedish International Development Agency Research support to Makerere University; And the European Union funded SPEED Initiative – Supporting Policy Engagement for Evidence based Decision-making for Universal Health Coverage in Uganda.
